# Fabrication and Optimisation of Ti-6Al-4V Lattice-Structured Total Shoulder Implants Using Laser Additive Manufacturing

**DOI:** 10.3390/ma15093095

**Published:** 2022-04-25

**Authors:** Oliver Bittredge, Hany Hassanin, Mahmoud Ahmed El-Sayed, Hossam Mohamed Eldessouky, Naser A. Alsaleh, Nashmi H. Alrasheedi, Khamis Essa, Mahmoud Ahmadein

**Affiliations:** 1 School of Engineering, University of Birmingham, Birmingham B152TT, UK; o.bittredge@bham.ac.uk (O.B.); k.e.a.essa@bham.ac.uk (K.E.); 2 School of Engineering, Technology, and Design, Canterbury Christ Church University, Canterbury CT1 1QU, UK; 3 Department of Industrial Engineering, Arab Academy for Science Technology and Maritime, Alexandria 21599, Egypt; m_elsayed@aast.edu (M.A.E.-S.); hossam.eldessouky@aast.edu (H.M.E.); 4 College of Engineering, Imam Mohammad Ibn Saud Islamic University, Riyadh 11564, Saudi Arabia; naalsaleh@imamu.edu.sa (N.A.A.); nhrasheedi@imamu.edu.sa (N.H.A.); maahmadein@imamu.edu.sa (M.A.); 5 Department of Production Engineering and Mechanical Design, Tanta University, Tanta 31512, Egypt

**Keywords:** additive manufacturing, laser powder bed fusion, lattice optimisation, Young’s modulus, orthopaedic implants

## Abstract

This work aimed to study one of the most important challenges in orthopaedic implantations, known as stress shielding of total shoulder implants. This problem arises from the elastic modulus mismatch between the implant and the surrounding tissue, and can result in bone resorption and implant loosening. This objective was addressed by designing and optimising a cellular-based lattice-structured implant to control the stiffness of a humeral implant stem used in shoulder implant applications. This study used a topology lattice-optimisation tool to create different cellular designs that filled the original design of a shoulder implant, and were further analysed using finite element analysis (FEA). A laser powder bed fusion technique was used to fabricate the Ti-6Al-4V test samples, and the obtained material properties were fed to the FEA model. The optimised cellular design was further fabricated using powder bed fusion, and a compression test was carried out to validate the FEA model. The yield strength, elastic modulus, and surface area/volume ratio of the optimised lattice structure, with a strut diameter of 1 mm, length of 5 mm, and 100% lattice percentage in the design space of the implant model were found to be 200 MPa, 5 GPa, and 3.71 mm^−1^, respectively. The obtained properties indicated that the proposed cellular structure can be effectively applied in total shoulder-replacement surgeries. Ultimately, this approach should lead to improvements in patient mobility, as well as to reducing the need for revision surgeries due to implant loosening.

## 1. Introduction

An orthopaedic implant is a medical device that is designed to restore the function of a damaged joint, bone, or cartilage by replacing the worn-out part. An example of orthopaedic implant is the total shoulder arthroplasty (TSA) which involves the replacement of the glenohumeral joint with an artificial prosthesis [1,2]. This prosthesis consists of an adjustable-length stem, which is inserted into the humeral (upper arm) bone, and a polished head that is introduced into the glenoid fossa of the scapula [3]. A problem that is commonly encountered with long-term bone implantation is the large discrepancy between the elastic modulus of the human bones (which range from 3 to 20 GPa) and that of the metallic implants, which are higher by about an order of magnitude [4,5]. Typically, the elastic moduli of Ti and stainless-steel alloys (biomaterials used extensively in implantation surgeries) are about 110 and 270 GPa, respectively [6,7]. The presence of an implant with a larger stiffness than those in human bones reduces the amount of load transferred to the adjacent bones. This phenomenon is known as stress shielding, and often results in the resorption of the bone and, subsequently, the loosening of the prosthesis, which causes the patients to require revision surgery [7,8]. Boileau et al. [9] reported that humeral loosening accounted for 21% of orthopaedic-implant revision surgeries. A typical solution for reducing this difference in Young’s modulus is to introduce a porous or cellular structure into the design of the implant [10,11]. Porous materials play a key role in bone and tissue replacements due to their relatively low stiffness and the enhanced osseointegration through bone cell ingrowth [12,13].

Cellular lattice structures are topologically ordered, trusslike structures formed of repeatable unit cells [14]. Examples of lattice structures include internal bone structure (cancellous bone), wood, foams, and honeycomb. Lattice structures are characterised by open pores and reticulated (nonstochastic) orientations of their constituent unit cells. Each unit cell is composed of a number of struts that are connected at specific nodes. The cell is usually characterised by strut dimensions and connectivity [15,16]. One of the main advantages of lattice structures is the ability to create parts with reduced mass, which enables the production of lightweight objects while still maintaining performance. In addition to their high relative porosity, lattice structures also have a high surface area compared to solid objects. Such superior characteristics make cellular materials ideal for several applications, including filtration, heat exchanging, and orthopaedic prosthesis [17].

Traditional manufacturing techniques such as punching, powder metallurgy, investment casting, and metal foaming processes are being widely used to fabricate cellular structures [6,18]. However, the produced cellular materials demonstrate a stochastic arrangement of either open or closed porosity (rather than an ordered porous structure) with a considerable variability in the shape and dimensions of the porosity [19]. This leads to anisotropic mechanical properties, which make the design of such structures more complicated [20]. Nonetheless, these inadequacies can prevail over advanced manufacturing methods such as additive manufacturing (AM) technologies due to their unique capabilities of producing complex shapes and geometries that cannot be produced by traditional manufacturing methods [21].

An important AM technique is laser powder bed fusion (LPBF), or selective laser melting (SLM), which uses a high-powered laser to melt a metal powder to form a solid area of the material [22,23]. This process continues layer-by-layer until it produces the finished component. LPBF can be used to fabricate parts made from various metals, including steel, aluminium, and titanium alloys [24,25]. There are many examples in the literature of the use of LPBF to produce complex porous and lattice structures for biomedical applications. Onal et al. [26] used LPBF to manufacture porous gradient structures comparable to the stiffness of bone while improving the biological properties of the structures. Burton et al. [27] also used LPBF to produce a number of different complex lattice shapes for orthopaedic hip spacers, which performed favourably when tested. Other authors have reported the design of lattice structures for different orthopaedic and biomedical applications, including hip and knee replacements, mandible and skull implants, as well as load-bearing implants [28]. Their main objective was to control the elastic modulus of the implant in such a way that minimised the stiffness mismatch between the implant and parent bones.

Recently, the design of cellular structures was further improved through the integration of AM technology and topology-optimisation strategies that allowed the design of lattices with improved specific strengths while minimising material usage. Topology optimisation is a structural optimisation technique that repeatedly optimises the material arrangement within a design space for a given set of loads and boundary conditions in order to maximise the system’s performance. Earlier studies by Li et al. [29], Panesar et al. [30], and Lui et al. [31] discussed different approaches to the application of different topology-optimisation tools to design functionally graded porous materials for biomedical applications. It was concluded that the graded cellular structures had experienced a superior performance compared to the uniform lattice structures. Topology optimisation was also used by He et al. [32] along with lattice optimisation to produce a solid-lattice hip prosthesis as a method of reducing stress shielding. It was found that the optimised implant could theoretically reduce stress shielding by over 50%. As opposed to the previous research, this study focused on utilising lattice structures to reduce the stiffness, rather than maximising it. Sutradhar et al., 2016 [33] also produced implants for the skull using topology optimisation, and stress shielding was one of the issues addressed.

Despite the reviewed research, the application of lattice-optimisation tools in the design of a humeral prosthesis’ internal structuring has still not been investigated in the literature. In addition, previous investigations related to the use of additive manufacturing for the fabrication of orthopaedic implants were mainly focused on knee and hip implants. There is a clear gap in the literature regarding the additive manufacturing of cellular shoulder prostheses. Therefore, and to highlight the potential of AM in shoulder implants, an optimisation of a humeral prosthesis’ internal lattice structuring was examined throughout this study. Due to its biocompatibility and suitability for orthopaedic applications, the Ti-6Al-4V alloy was selected for this study. LPBF was used to manufacture solid and lattice cubes due to its unique capability of producing small strut diameters down to 0.5 mm. Lattice-optimisation tools were applied, with the intention of reducing both the weight and stiffness of a humeral stem implant while increasing its functionality. The performance of the proposed lattice structure was assessed by using finite element analysis, as well as mechanical testing. This allowed the design and fabrication of lattice structures with controlled properties that could be effectively used for humeral stem replacement surgeries.

## 2. Methodology

### 2.1. Design and Modelling

**Figure 1:**
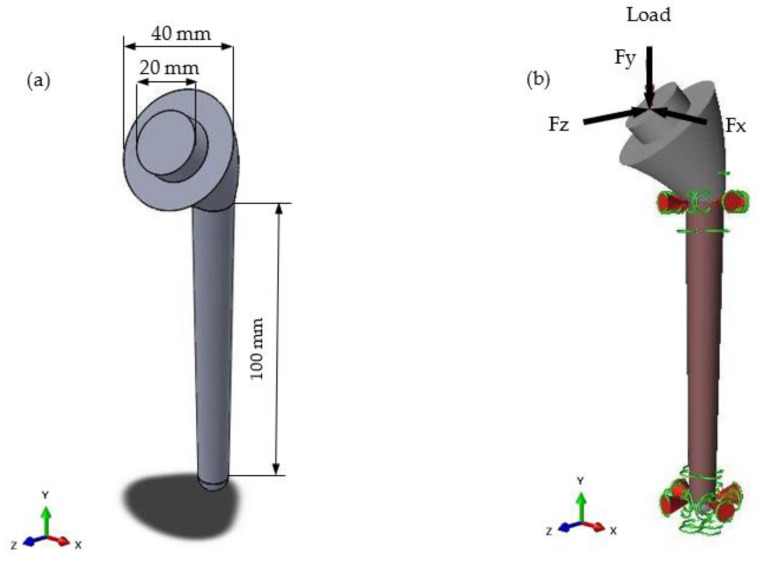
(**a**) CAD geometry of humeral implant stem design created in Solidworks 2018; (**b**) implant geometry showing applied load and supports. Highlighted area represents the design space. Markers at the base and neck represent the supports. Arrow at the head represents loading.

The geometry of the humeral implant was created in Solidworks 2018 (Dassault Systems, Velizy-Vilacoublau, France) based on humeral geometry and measurements described by Pearl [34]. Figure 1a shows the implant’s computer-aided design (CAD) geometry. A standard stem length was chosen to ensure enough design space to create the lattice structures. The design was kept simple to reduce complications during the lattice-optimisation step.

The model was imported into Altair Inspire 2018 (Altair, Troy, MI, USA) to perform the lattice optimisation. The first step was to apply the load and supports. The supports (as seen in Figure 1b) represented an interference fit, which is the best fixation method for improving osseointegration (an important functionality of a lattice structure for biomedical implants) [35]. This is shown as fixed boundary conditions in the upper and lower part of the stem implant. The load on a humeral implant will vary depending on the activity. Bergmann et al. [36] used an implant fitted with a strain gauge to measure the joint’s contact forces in vivo. Two load cases were chosen from this data to represent two common movements of the shoulder joint. These are displayed in Table 1. The highlighted region in Figure 1b represents the design space, which is the area in which the lattice structures were generated. The upper region of the implant was not included in the design space to maintain its stiffness as a design requirement [32].

**Figure 2:**
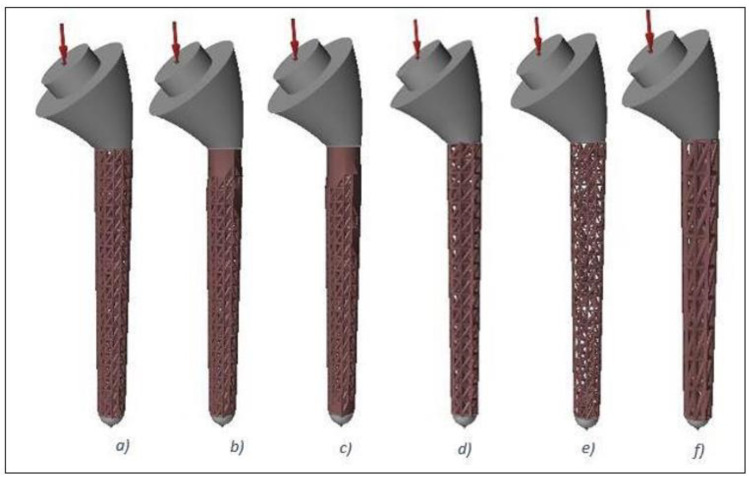
Humeral stems with lattice structures produced using lattice optimisation. From left to right: (**a**) Lattice 1; (**b**) Lattice 2; (**c**) Lattice 3; (**d**) Lattice 4; (**e**) Lattice 5; and (**f**) Lattice 6, corresponding to the parameters described in Table 2.

A ‘minimise mass’ optimisation objective was chosen during the lattice-optimisation setup, and three different parameters were changed to achieve six different face-centred cubic (FCC) lattice designs. These parameters were: strut length, strut diameter, and percentage of lattice in the design space. The percentage of lattice in the design space was altered to create graded lattice-solid structures for the first three optimisations. The fourth, fifth, and sixth optimisations changed the strut length and strut diameter. The values chosen for the parameters and their corresponding lattices are given in Table 2. Each of these lattices was then generated in Altair Inspire software, and are shown in Figure 2.

**Figure 3:**
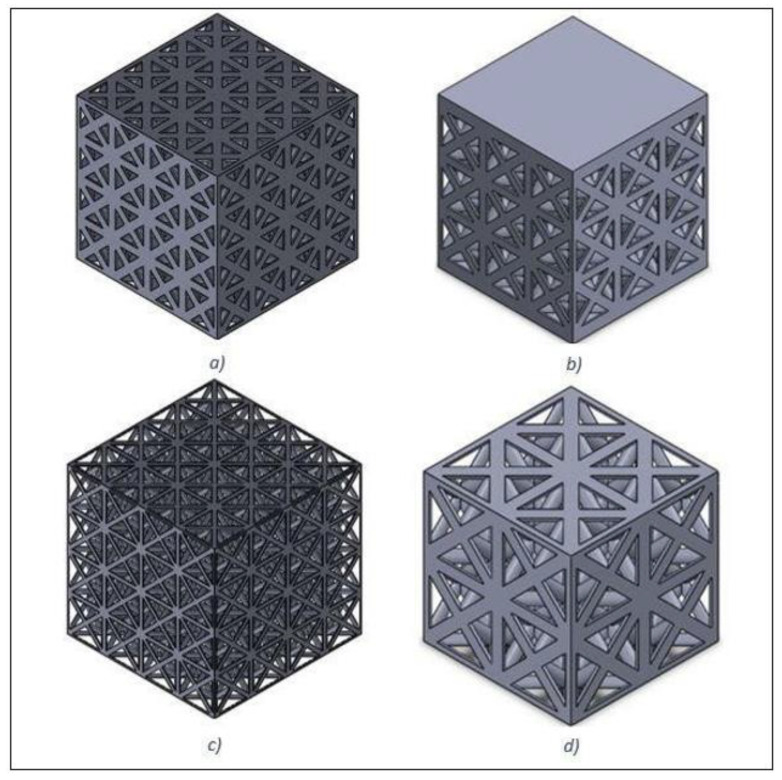
CAD models created in Solidworks 2018 of compression test samples representing face-centred cubic lattice structures of (**a**) Lattice 1; (**b**) Lattice 4; (**c**) Lattice 5; and (**d**) Lattice 6.

Altair Inspire’s analysis tool was used to evaluate the lattice structures for each load case and give an idea for values such as maximum deflection, maximum von Mises stress, and minimum safety factor. Another value that was recorded was the resultant mass of each optimised implant. This was to confirm whether the lattice structures succeeded in making the implant more lightweight or not. FEA was used to further analyse the lattice structures described above by simulating a simple compression test. From this, the theoretical Young’s modulus of each structure could be measured to determine whether there was a significant reduction. The first step was to create the compression test samples for each lattice structure. Solidworks 2018 was used to create the samples at 20 × 20 × 20 mm^3^ [30], which comprised repeating unit cells derived from the lattice structures (with different strut lengths and strut diameters presented in Table 2) that had been created in Altair Inspire. As samples 1, 2, and 3 had the same strut lengths and diameters, only one was selected to create the compression samples. Figure 3a–d show the CAD models of Lattices 1, 4, 5, and 6, respectively.

Each sample was imported into Abaqus 2017 (Dassault Systemes, Velizy-Vilacoublau, France) to conduct FEA, with C3D8R quadratic elements chosen for the lattice structure that was 1 mm in size. The material properties of the Ti-6Al-4V titanium alloy were obtained through the experimental testing of fabricated solid compression samples. A discrete rigid, 2D planar part using R3D4 elements was created to represent the top plate during a compression test, and an assembly was created consisting of the sample and the plate. A mesh sensitivity analysis was carried out to optimise the element size and computational time at which an accurate solution could be achieved.

**Figure 4:**
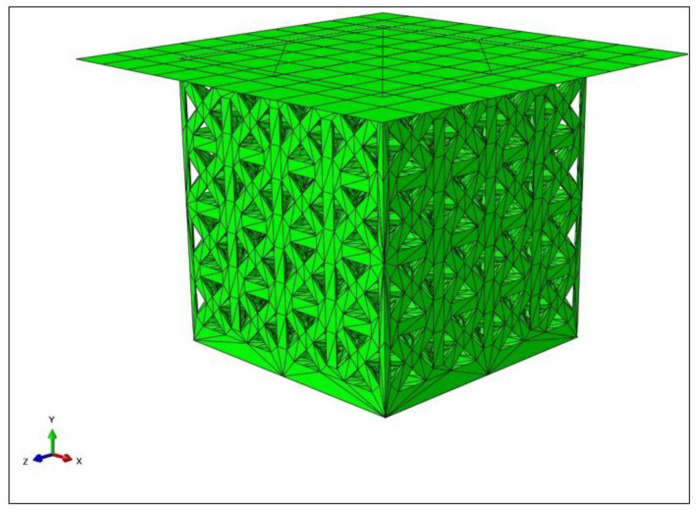
Assembly of plate and Lattice 1 sample as set up in Abaqus.

Figure 4 shows the assembly of the plate and one of the samples (lattice from experiment 6 in Table 2) in Abaqus.

Next, the boundary conditions (BCs) and loads were applied. The bottom of the sample was fixed using an Encastre BC, which restricted all degrees of freedom (DOFs). All DOFs were restricted for the top plate apart from its displacement in the y-axis. A surface-to-surface contact was applied between the top plate and the top of the sample, and a coefficient of friction of 0.3 was defined between the surfaces. A displacement load was applied, covering 10 mm in the negative y-axis to represent a compression test. A static general step (to imitate a static compression test) was used, and a reference point was created on the top plate to record the reaction force and displacement. The assembly was then meshed using second-order C3D10 tetrahedral elements. The simulation was run for each sample, and 100 data points for the reaction force and displacement were extracted. The data were then plotted to obtain engineering stress–strain diagrams for different lattice designs.

### 2.2. Fabrication and Characterisation

**Figure 5:**
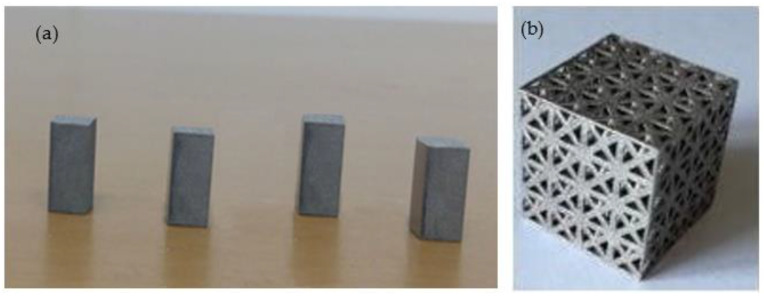
Ti-6Al-4V samples fabricated using LPBF: (**a**) LPBF coupons for compression-testing properties; (**b**) optimised lattice design.

Ti-6Al-4V gas-atomised alloy powder by LPW Technology and supplied by TLS Technik GmbH was used. The majority of the powder particle sizes ranged between 19 and 45 µm. A laser diffraction analyser (Microtrac) following the ASTM B822 standard was used to measure the powder particle sizes. All samples were fabricated using a Renishaw RenAM 500M Additive Manufacturing system (Renishaw plc, Wotton-under-Edge, UK). The samples were produced using standard process parameters for Ti-6Al-4V: a laser power of 200 W, a scanning speed of 1200 mm/s, and a layer thickness of 20 microns. The samples were produced on a titanium plate and under argon control down to O_2_ < 100 ppm. The elastic and plastic properties of the additively manufactured bulk Ti-6Al-4V alloy were first obtained by producing solid compression samples with rectangular cross-sections of 6 × 6 mm and a height of 12 mm (see Figure 5a). The obtained material properties were then used in the FEA model. In another build, the optimised FCC lattice structures were also fabricated using the aforementioned conditions and were validated against the FEA model (Figure 5b). All samples were cut off the building substrate using EDM, ultrasonically cleaned in acetone for five minutes to remove lightly bonded particles, and then dried with compressed air. Prior to characterisation, the as-fabricated lattice structures and the chemical compositions of the samples were examined using a Hitachi tabletop scanning electron microscope.

**Figure 6:**
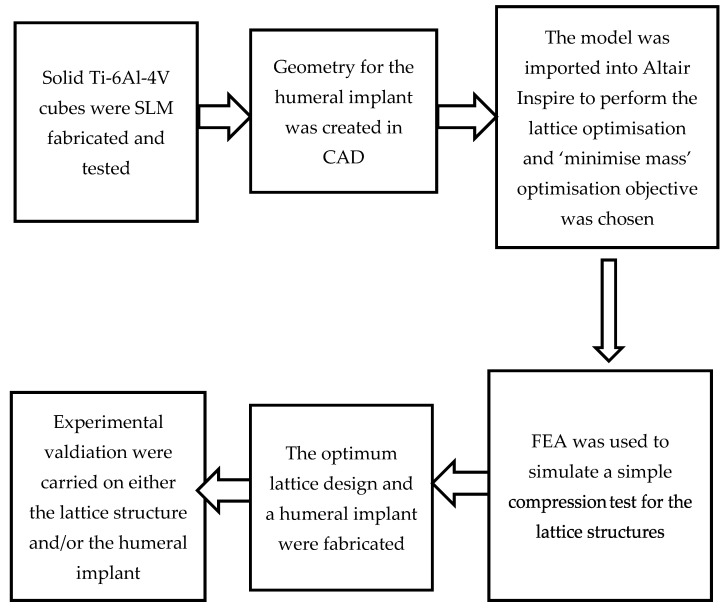
Workflow of performed computational and experimental testing in the current study.

A scanning electron microscope (SEM) was used to observe any deviation between the CAD structures and the manufactured samples. The fabricated test coupons, as well as the optimised lattice structure, were tested in a static compression using an ESH 200 kN Servo-Hydraulic Universal Testing Machine at a rate of 0.1 mm/s. The compression test’s resulting stress–strain curves were analysed to determine the mechanical properties of both the solid material and lattice structure fabricated via LPBF. The surface roughness of the printed prototype implant was measured with a Talysurf 120L surface profilometer from Taylor Hobson (with a resolution of 12.8 nm at 10 mm). The arithmetic mean surface roughness (Ra) was used to describe the parts’ surface qualities. Two measurements were carried out at the top head of the implant; the average value of these two measurements was considered to express the surface roughness of the implant. Finally, Vickers microhardness measurements were performed at the head of the prototype using a 100 g load and an indent time of 15 s. The measurements were carried out at two separate lines, and five measurements were recorded on each line. Then, the average was calculated. A block diagram illustrating the workflow of the performed computational and experimental testing is presented in Figure 6.

## 3. Results and Discussion

**Figure 7:**
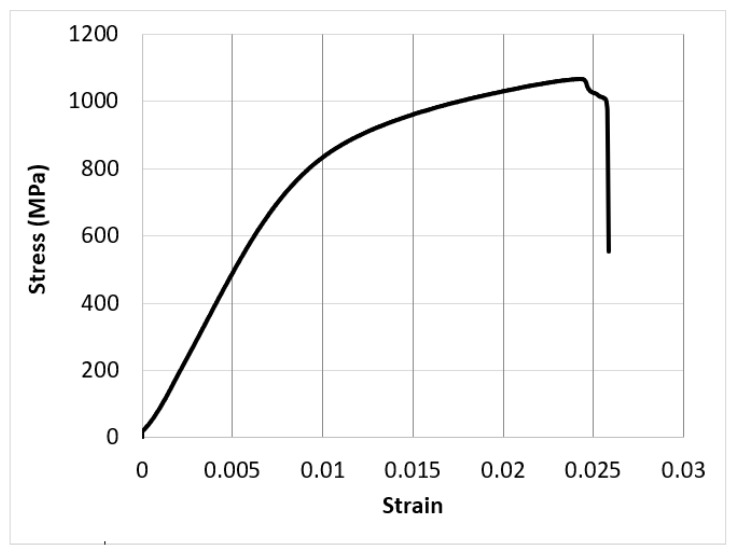
A fracture compression test results of a Ti-6Al-4V sample fabricated using LPBF.

Figure 7 shows an experimental stress–strain diagram for one of the Ti test coupons fabricated via LPBF. The average and standard deviation of the elastic modulus, yield strength, compressive strength, and ductility of the three samples were 102 ± 5 GPa, 920 ± 15 MPa, 1170 ± 20 MPa, and 2.6 ± 0.2%, respectively.

The mechanical properties of the additively manufactured coupons were used to simulate the six lattice designs when loaded by using load cases one and two (as described in Table 2). The modelling results from the Altair Inspire software analysis are given in Table 3. It was clear that adopting the lattice design successfully decreased the weight of the implant. The results showed a significant reduction in the maximum deflection values in the partially solid implants (Lattices 2 and 3) compared to the other lattice structures. In addition, the load cases did not affect the implant’s resultant mass reduction, and it can be noted that Lattice 5 was the lightest design, which showed advantages, as they promoted body tissue growth and the implant’s osseointegration.

**Figure 8:**
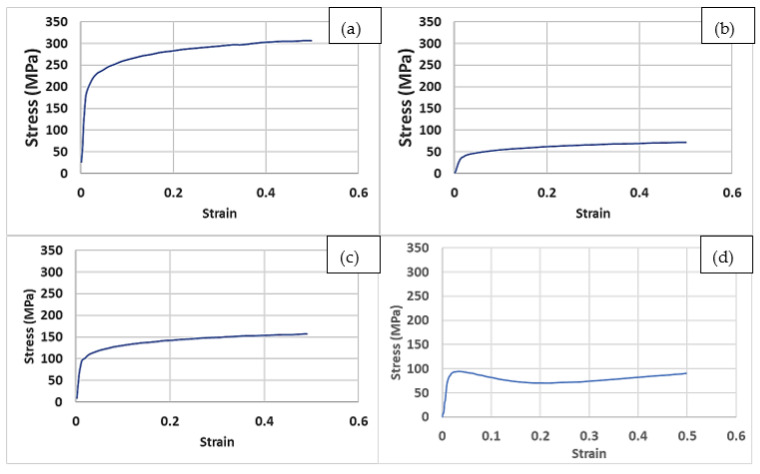
Stress vs. strain curves obtained from FEA: (**a**) Lattice 1; (**b**) Lattice 4; (**c**) Lattice 5; (**d**) Lattice 6.

As mentioned earlier, Lattices 1, 2, and 3 had the same strut lengths and diameters, so only one was used for the further compression analysis, and was noted as Lattice 1. The reaction forces and displacement data extracted from FEA of the four compression lattice cubes were used to plot the compressive stress–strain diagrams for different lattice structures (see Figure 8). From these graphs, the yield strengths and Young’s moduli were also obtained. The gradient, or slope, of the linear (elastic) region of the graph was used to calculate the Young’s modulus for each test, and the yield stress was obtained using the 0.2% strain-offset method [37]. The values obtained for different structures are shown in Table 4. The results showed a significant reduction in the Young’s modulus for all samples. The figures show a clear correlation between the reduction in mass and both the Young’s modulus and the yield strength. A higher mass reduction due to a high porosity content resulted in a lower stiffness and a lower yield strength. Similar reductions in the Young’s moduli when using porous and lattice structures were reported in the literature. For their nickel–titanium porous structures, Bandyopadhyay et al. [18] obtained Young’s moduli between 2 and 18 GPa. In addition, a small modulus of 1.7 GPa was obtained by Krishna et al. [38] from their porous titanium structures. This study further supported the application of lattice structures to produce orthopaedic implants with a low stiffness.

In addition, the surface area/volume ratios were calculated in Solidworks for different lattice designs, and are presented in Table 4. A high surface area/volume ratio is key to improving the osseointegration of an implant [39]. From these results, it was observed that Lattice 5 had the highest ratio, at 8.36 mm^−1^.

Given its high strength and excellent biocompatibility, Ti-6Al-4V is a preferred candidate for biomedical implants and scaffolds using PBF. As described above, the properties of the implant should, to a great extent, mimic those of the host bone and surrounding tissues. For this reason, a fully solid Ti-6Al-4V is unsuitable due to its impermeability, which prohibits the transport of body fluids and medication. In addition, the elastic modulus of Ti-6Al-4V (≈114 GPa) is much higher than that of human bones (which range from 3 to 20 GPa) [40,41]. This results in stress shielding of the bone tissue, and ultimately could result in the failure of the implant [42]. These problems were addressed here using the application of the lattice-optimisation tool and FEA modeling in the design of a biomedical implant that could integrate both the adequate mechanical strength and porosity to allow the orthopaedic implant to have both a sufficient strength and elastic modulus to withstand the applied stresses and the appropriate porosity to assist the flow of body nutrients, and would allow the encompassing tissues to grow inside the implant, which would improve its interfacial bonding with the natural bone. The FEA results (presented in Table 4) suggested that the examined lattice structures had reduced Young’s moduli. Lattices 1, 4, 5, and 6 had exhibited a modulus of 13.6, 6.3, 2.6, and 5.9 GPa, respectively. Except for Lattice 5, the moduli of all other designs fell within the 3–20 GPa range for bones.

To avoid plastic deformation and enhance functional stability, a medical implant should have a high yield strength. High strength is also needed in order to prevent spring-back during and after the surgical procedure [43]. Morgan et al. reported that the compressive yield strength of human cortical bones ranged from 100 to 130 MPa [44]. Therefore, Lattice 1 was the best design, as it combined the highest yield strength (of 200 MPa), which fairly exceeded that of human bones, and an acceptable Young’s modulus (of 13.4 GPa). The suitability of Lattice 1 was further supported by its surface area/volume ratio of 3.71 mm^−1^, which was the second-highest ratio of the four designs, whereas Lattice 5 had a surface area/volume ratio of 8.36 mm^−1^. This high surface area/volume ratio suggested that this design would be more effective at improving osseointegration.

**Figure 9:**
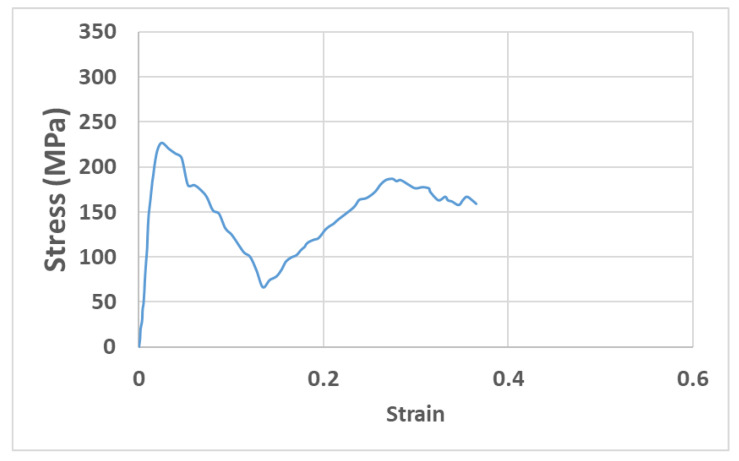
Fracture compression test results for a Ti-6Al-4V LPBF lattice structure with design dimensions of Lattice 1.

In order to validate the FEA predictions, a Ti-6Al-4V lattice structure of strut length, strut diameter, and percentage of lattice in a design space of 5 mm, 1 mm, and 100 (design dimensions of Lattice 1), respectively, was fabricated using LPBF, and was then characterised as described above in Section 2.2. The fabricated lattice’s compressive yield strength and elastic modulus were found to be 200 MPa and 11.8 GPa, respectively. The stress–strain diagram of the experimentally tested lattice sample (of design No. 1) is given in Figure 9. By comparing the yield strength and the elastic modulus of the compression test sample (Figure 9) and of the FEA model (Table 4) for the same lattice design (No. 1), the respective errors in the FEA model prediction were found to be about 0 and 13%, suggesting an acceptable accuracy of the model in estimating the mechanical properties of the proposed lattice design.

Dumas et al. 2017 [45] observed an up to 40–50% difference between their FEA and experimental results, and stated that this could have been due to deviations in the geometry between the CAD model and the fabricated sample. It could be stated that the irregularities could reduce the mechanical properties, including the stiffness and strength. Any deviations, even small ones, in the strut diameter between the printed lattice and that defined in the CAD model, the partially melted powder particles, as well as the presence of porosities, can cause deviations between the FEA and experimental results.

**Figure 10:**
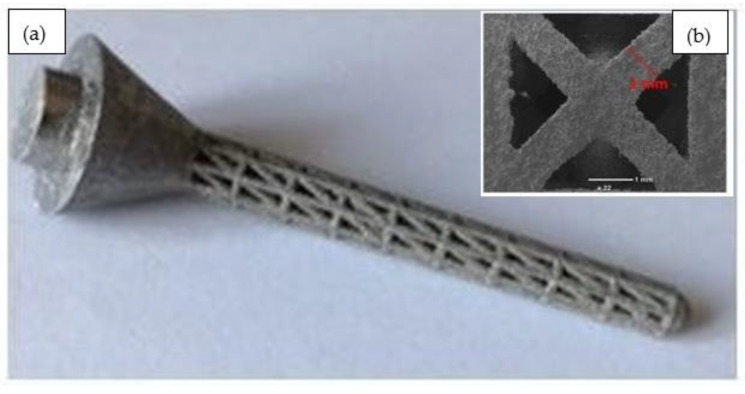
(**a**) Humeral implant containing a lattice structure; (**b**) SEM image of the manufactured Ti lattice structure.

The Ti-6Al-4V humeral implant containing a lattice structure in the stem was also fabricated according to the design of Lattice 1, as shown in Figure 10a. The SEM morphology of the strut surface of the as-built Ti lattice structure shown in Figure 10b indicated the same original strut diameters as defined in the CAD. This revealed a good consistency between the CAD model and the printed structure. The struts of the lattice structures were found to be solid, connected, and continuous, indicating that the powder melted well throughout the LPBF process. Any slight variations between the CAD design and the printed struts may have been due to the laser’s power fluctuations during the LPBF process and/or the buildup of powder particles in some areas. The SEM results indicated that the lattice was manufactured successfully with no visible defects or broken cells, confirming the capability of LPBF to reproduce the cellular lattice structures according to the optimised design. The surface chemical composition of the samples was measured using EDS analysis. Table 5 lists the average values of the SEM-EDS measurements of the samples, which determined the elemental composition on the sample’s surface. The measurements were in agreement with the typical alloy composition, although the oxygen level was slightly higher than the typical values.

**Figure 11:**
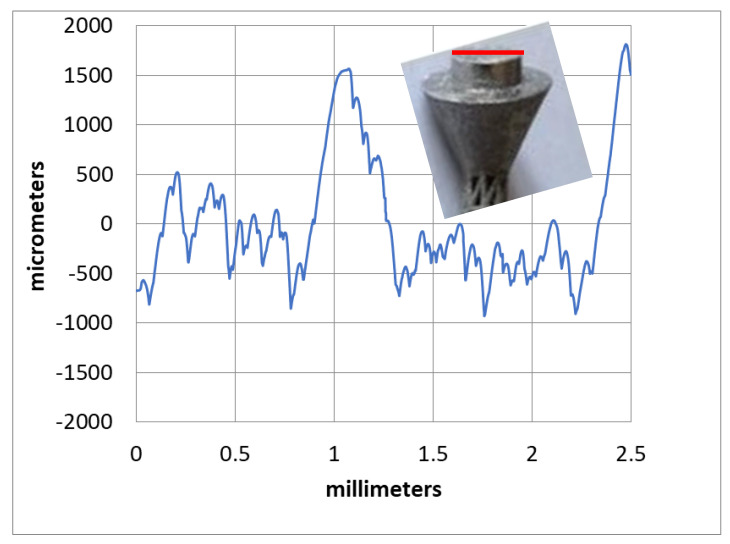
Typical surface roughness profile through the highlighted line (in red) at the head of the implant.

The measured surface roughness profile at the top head of the implant is shown in Figure 11. The arithmetic mean surface roughness (Ra) was found to be 9.3 µm. It was suggested that an implant should have enough surface roughness to allow human tissues to grow into it. Textured implant surfaces have more surface area for bone integration via the osseointegration process than smooth surfaces. Earlier studies recommended that a surface roughness of 1 to 10 microns would be required to enhance both the osteoconduction (inmigration of new bone) and osteoinduction (new bone differentiation) processes [46]. Therefore, the fabricated implant prototype’s surface roughness (9.3 µm) was within the appropriate range for medical implants.

**Figure 12:**
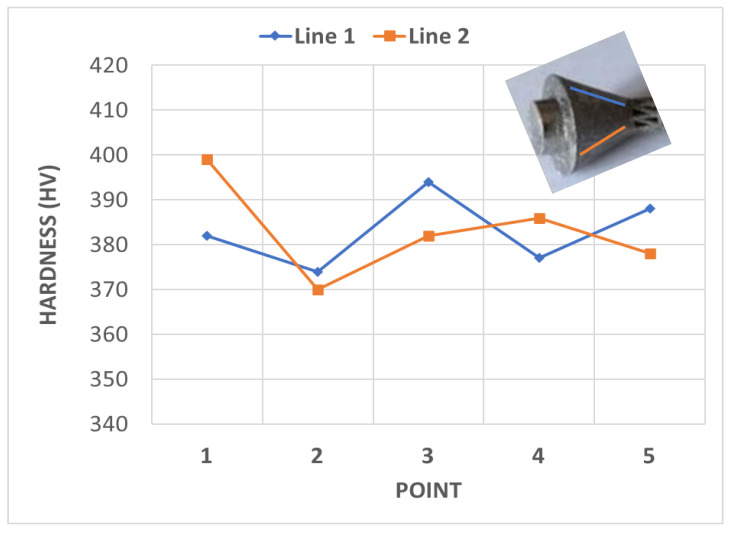
Results of hardness analysis at the two lines highlighted at the head of the implant.

The average hardness of the printed implant prototype was determined to be 383 HV, as shown in Figure 12, which was equivalent to the range of hardness values for bulk material in the literature, which varied from 340 to 395 HV [47]. In addition, the hardness results for the current study were in accordance with those reported by Khorasani [48], who obtained an average hardness of about 390 HB (≈412 HV) for Ti-6Al-4V samples produced using SLM with process parameters set to achieve a 98% relative density.

Measuring hardness is a possible way to assess the mechanical properties of bone tissue. It has been found that hardness can be considered as one of the characteristics that determines the quality of bone tissue [49]. According to Bouxsein, bone quality can be defined as the ability of a bone to resist fracture [50]. The author stated that the skeleton has many functions, including allowing for locomotion, protection of vital internal organs, and assisting in mineral homeostasis and hematopoesis. However, if a bone is broken, it can fulfill few, if any, of its many functions. Earlier studies also suggested that the degree of mineralisation of bone tissue strongly depends on the bone’s hardness [51]. The hardness of the printed implant in the current study was measured to be about 380 HV, which was much higher than that of human cortical bones (about 42 HV) [49]. This would decrease the incidence of wear of the implant material, and ultimately ensure superior performance of the implant.

Although the current methodology obtained the required mechanical properties in terms of strength, Young’s modulus, hardness, mass reduction, and expected improved body-fluid transportation through the lattice structures, other properties, such as fatigue, can be determined to investigate how the implant would behave under unsteady loading conditions. Further use of topology and lattice-optimisation tools can be employed to design solid-lattice implant structures for humeral prostheses. Different types of unit cells (e.g., Schwartz primitive, diamond, and cylinder grid) can be evaluated and compared. In vitro tests could also be conducted to further study the extent of osseointegration due to the lattice structures. Most of the current literature concentrated on load-bearing implants such as hip and knee prostheses; therefore, it would be beneficial to investigate lattice structures used in a greater variety of orthopaedic applications.

## 4. Conclusions

This paper described the application of topology optimisation and FEA to optimise the lattice structure of a humeral implant used in total shoulder arthroplasty. The key findings of the study are listed below:

1. Implementation of the lattice design significantly decreased the implant weight by up to 44% compared to a fully solid implant.
2. The FEA results suggested Young’s moduli for the examined lattice structures of between 2 and 13 GPa, which was comparable to that of human bones.
3. The experimental results showed that an LPBF-fabricated Ti-6Al-4V lattice structure with a 5 mm strut length, 1 mm strut diameter, and 100% lattice in the design space had a yield strength of 200 MPa, an elastic modulus of 11.8 GPa, a hardness of 380 HV, a surface roughness of 9.3 µm, and a surface area/volume ratio of 3.7 mm^−1^. These properties were suggested to be suitable for orthopaedic structures with a stiffness close to that of human bones and for improved bone ingrowth characteristics.

## Figures and Tables

**Table 1:** The two load cases used in the FEA model. The values for resultant forces and x, y, and z components are given.

Load Case	Type of Movement	Force in x-Direction (Fx)/N	Force in y-Direction (Fy)/N	Force in z-Direction (Fz)/N	Resultant Force (F)/N
Load Case 1	75° abduction	245.25	−725.94	−333.54	835.69
Load Case 2	120° flexion	225.63	−1049.67	−500.31	1184.49

**Table 2:** Six face-centred cubic lattice topologies with optimised geometries and their corresponding parameters.

Lattice No.	Strut Length (mm)	Strut Diameter (mm)	Percentage Lattice in Design Space
Lattice 1	5	1	100
Lattice 2	5	1	80
Lattice 3	5	1	60
Lattice 4	6	1	100
Lattice 5	5	0.5	100
Lattice 6	10	1.5	100

**Table 3:** Lattice structure performance in terms of maximum deflection, reduction in mass, and maximum von Mises stress when subjected to load cases 1 and 2.

Load Case	Design	Reduction in Mass %	Maximum Deflection (mm)	Maximum von Mises Stress (MPa)
1	Lattice 1	26.6	0.1006	82.59
	Lattice 2	27.8	0.0408	37.91
	Lattice 3	28.5	0.0404	37.81
	Lattice 4	34.7	0.1685	130.2
	Lattice 5	43.5	0.3615	259.7
	Lattice 6	24	0.1072	83.37
2	Lattice 1	26.6	0.1508	122.8
	Lattice 2	27.8	0.0607	57.09
	Lattice 3	28.5	0.0601	56.94
	Lattice 4	34.7	0.2526	193.8
	Lattice 5	43.5	0.5421	389.6
	Lattice 6	24	0.1607	125.2

**Table 4:** FEA results for the mechanical properties and surface area/volume ratios of different lattice structures.

Lattice Design	Yield Strength (MPa)	Elastic Modulus (GPa)	Surface Area/Volume Ratio (mm^−1^)
1	200	13.4	3.71
4	96	6.3	3.40
5	40	2.6	8.36
6	90	5.9	2.99

**Table 5:** EDS analysis of the fabricated implant.

Ti	V	Al	O
88.55	4.75	6.45	0.25

## Data Availability

Not applicable.
